# Hybrid Carbon Microfibers-Graphite Fillers for Piezoresistive Cementitious Composites

**DOI:** 10.3390/s21020518

**Published:** 2021-01-13

**Authors:** Hasan Borke Birgin, Antonella D’Alessandro, Simon Laflamme, Filippo Ubertini

**Affiliations:** 1 Department of Civil and Environmental Engineering, University of Perugia, via Goffredo Duranti 93, 06125 Perugia, Italy; hasanborke.birgin@unipg.it (H.B.B.); antonella.dalessandro@unipg.it (A.D.); 2 Department of Civil, Construction and Environmental Engineering, Iowa State University, Ames, IA 50011, USA; laflamme@iastate.edu

**Keywords:** smart-materials, hybrid fillers, graphite, carbon microfibers, strain sensing, piezoresistivity, conductivity, self-monitoring

## Abstract

Multifunctional structural materials are very promising in the field of engineering. Particularly, their strain sensing ability draws much attention for structural health monitoring applications. Generally, strain sensing materials are produced by adding a certain amount of conductive fillers, around the so-called “percolation threshold”, to the cement or composite matrix. Recently, graphite has been found to be a suitable filler for strain sensing. However, graphite requires high amounts of doping to reach percolation threshold. In order to decrease the amount of inclusions, this paper proposes cementitious materials doped with new hybrid carbon inclusions, i.e., graphite and carbon microfibers. Carbon microfibers having higher aspect ratio than graphite accelerate the percolation threshold of the graphite particles without incurring into dispersion issues. The resistivity and strain sensitivity of different fibers’ compositions are investigated. The electromechanical tests reveal that, when combined, carbon microfibers and graphite hybrid fillers reach to percolation faster and exhibit higher gauge factors and enhanced linearity.

## 1. Introduction

Structural Health Monitoring (SHM) is the automation of the condition assessment process, aimed at increasing structural safety and improving maintenance and repair operations [1]. SHM is conducted by collecting measurements that can be processed into actionable information. Available off-the-shelf sensors that can be deployed over large structural systems possess some drawbacks: (i) they are typically expensive [2]; (ii) their integration within the structural system requires high technical expertise [3]; (iii) their durability and robustness are limited; and (iv) they require a certain level of inspection and maintenance [4].

To alleviate challenges associated with common SHM sensors, multifunctional materials have been proposed, capable of self-sensing [5]. A popular method to induce self-sensing capability is to build on the piezoresistive effect [6,7], namely the capacity of the electrical resistance to react to mechanical stress, enhanced by the introduction of conductive fillers [8,9]. Of interest to this paper are cement-based matrices, for which it is possible to disperse conductive fillers within the matrix to yield a desirable piezoresistive behavior [10]. The amount of fillers necessary to achieve such piezoresistivity is often slightly above the electrical percolation threshold, namely the region where the material’s electrical behavior transits from an insulator to a conductor. However, excessively exceeding the percolation threshold may result in high electrical noise and poor signal-to-noise ratios. The percolation threshold depends on the type and morphology of the filler and is generally determined through experimental tests [11,12] or micromechanical modeling [13]. Among common conductive fillers, carbon nanotubes are reported to percolate between 0.75% and 1% weight ratio to the cement [14], and graphite around 20%: this difference is due to their different aspect ratio. [15,16,17].

An advantage of self-sensing structural materials over traditional techniques is in the enhanced mechanical bonding [18,19] and durability [20,21]. They are also known to yield better sensing performance resulting from the significantly enhanced piezoresistive effect. For instance, literature shows that the gauge factor of cement composite fabricated with carbon-based fillers ranges from 250 (graphene nano platelets-cement mixture) to 4000 (carbon nanotubes-cement mixture) [22]. Some challenges associated with self-sensing or smart cement composites include the high costs of some fillers, in particular for nanosized carbon inclusions (e.g., carbon nanotubes, carbon nanofibers, graphene nanoplatelets), their tendency to agglomerate thus causing bad dispersion, and their possible negative effects on environment and health. These challenges importantly limit the scalability of the technology and their use in construction applications.

Hybrid fillers show promise in alleviating such issues. The combination of different types of fillers may create a synergistic effect, therefore necessitating lower doping levels. They may also yield better mechanical capacity [23], decreased costs, and reduced environmental and health impacts from the reduction of the required amount of filler. Literature reports studies on hybrid doping, including carbon black-graphene [24], carbon nanotubes-graphene nanoplatelets [25], carbon nanotubes-nano carbon black [26], carbon nanotubes-carbon fibers [27], carbon black-conductive rubber fibers [28], carbon black-polypropylene fibers [29], steel fibers-carbon nanotubes [30], carbon black-block co-polymer (SEBS) [31].

In this study, the combination of graphite (G) with carbon microfibers (CMF) for fabricating strain sensing cement matrix composites is studied. The aim of the study is to explore and assess strain sensing performance of combinations of hybrid fillers within a practical domain. Unlike hybrid combinations reported in the literature, both fillers are easy to disperse and the final material is scalable. Therefore, the mix design is suitable for in-situ production. As a matter of fact, CMF highly contribute to increasing the conductivity of the cementitious matrix and therefore accelerate electrical percolation. An analytical surface model of resistivity is developed to verify the positive influence of carbon fibers inside the cement matrix and identify the percolation of G+CMF hybrid fillers. The best performing material formulation in terms of output signals is also identified.

The rest of the paper is organized as follows. Section 2 introduces the materials and mixture characteristics. Section 3 presents the methodology, including a description of the sample geometries and the experimental setup for the electromechanical tests. Section 4 develops the analytical surface modeling of the samples’ resistivity. Section 5 presents results of the electromechanical tests. Section 6 concludes the paper.

## 2. Materials and Samples

**Figure 1:**
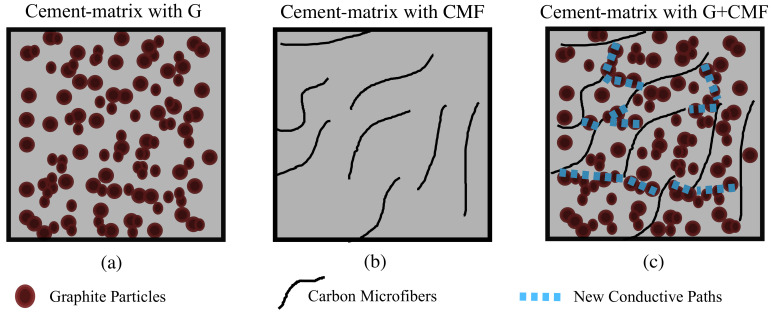
Illustration of accelerated percolation using hybrid filler: (**a**) G only; (**b**) CMF only; and (**c**) hybrid G+CMF filler showing a formed conductive path.

The hybrid inclusions considered in this paper consist of G and CMF (SIGRAFIL by SGL Carbon [32]). The cement matrix of the composite is fabricated using Portland cement (42.5R) and tap water. Materials properties are listed in Table 1. The mechanism accelerating percolation through the hybrid filler is illustrated in Figure 1, where it is noted that CMF are used as partial substitutes of G within the composite. Owing to their larger aspect ratios, CMF activate electrically conductive paths through the G particles well before they become connected among each other.

**Figure 2:**
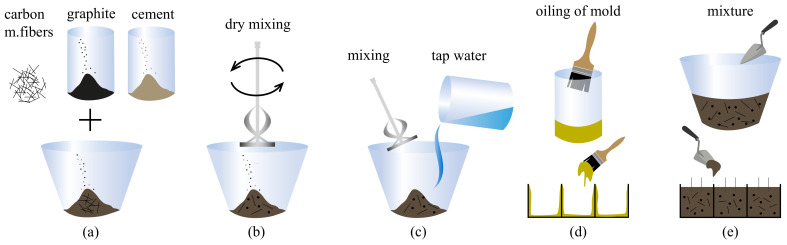
Preparation steps for cubic samples with G+CMF hybrid inclusions; (**a**) combination of CMF, G, and cement; (**b**) dry mechanical mixing of materials; (**c**) addition of water and mixing until homogeneity; (**d**) preparation and oiling of metal molds; and (**e**) the pouring of compound into molds and placement of steel electrodes.

The preparation steps for cubic samples of cement G+CMF composites are illustrated in Figure 2. The cement powder, graphite, and carbon microfibers were mixed together in their dry forms. After good dispersion was attained, water was added slowly to the compound and mixed until homogeneity. Unlike most nanosized inclusions, the selected types of graphite and carbon microfibers are easy to disperse in water, in particular following our mechanical mixing procedure [32]. This was confirmed by visual inspection of the water-filler suspensions, by optical microscope investigations of the hardened material, and by verifying repeatability of macroscopic electrical properties of nominally identical specimens, as discussed later. Later, the compound was poured into oiled molds followed by placement of steel net electrodes in their designated positions. All samples were cured in laboratory conditions. The sets of samples have six levels of CMF inclusions, starting at 0.2 g and doubling the weight subsequently. Each CMF level includes a subset of samples of different graphite-to-cement weight ratios. The sample sets also includes graphite-only inclusions. The fabrication of samples showed that a 0.25% CMF-to-cement weight ratio is an upper limit governed by sufficient workability. Twenty-four different types of samples were fabricated with different loading of G and CMF, with three samples fabricated per type, for a total of 72 samples. Their individual constitutions are listed in Table 2, including the amounts of materials in terms of weight and weight to cement ratio (G/c and CMF/c), volumetric fractions (*v*), and water-to-cement ratio (w/c).

**Figure 3:**
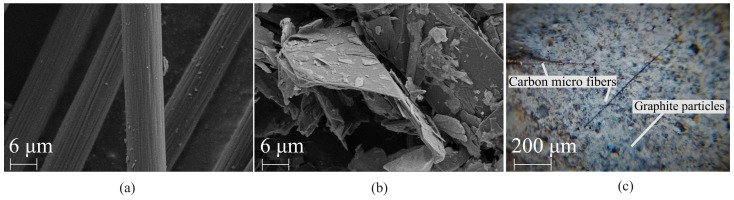
Micrographs of (**a**) CMF; (**b**) G; and (**c**) cement matrix for sample 1-32CMF10G showing the dispersion of both fillers.

Figure 3a,b show the SEM micrographs of G and CMF, respectively highlighting their difference in aspect ratios. Figure 3c is a micrograph obtained by optical microscope of the cement composite with sample 1-32CMF10G showing their good dispersion in the same material matrix. The figure also evidences the difference in sizes between a microfiber and a graphite particle. Remark that due to the relatively large sizes of the adopted inclusions, a simple optical microscope provides the necessary magnification to assess quality of filler dispersion, without requiring an SEM inspection. The samples prepared with the selected combinations of fillers were electrically tested for characterizing the electromechanical model and identifying the best typologies for sensitivity tests.

## 3. Electromechanical Model and Measurement

**Figure 4:**
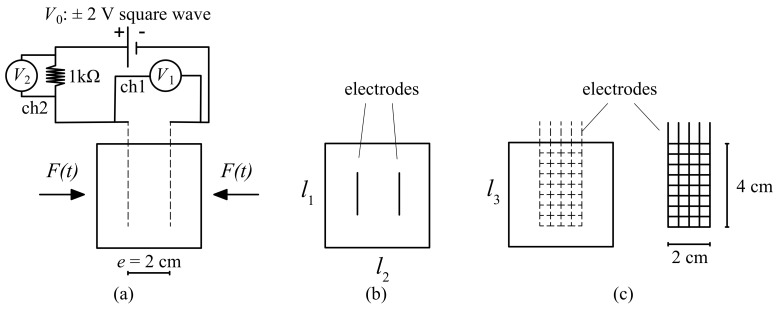
The sample and electromechanical setup illustration; (**a**) general view of the cube samples together with electrodes, electric circuit with channels and voltage readings, and, the applied load F(t); (**b**) the top view of the sample and the mutual distance of electrodes; (**c**) side view of sample and electrodes.

For the development of the electromechanical model, the geometry schematized in Figure 4 has been considered, where $l_{i}=5$ cm is the length of the fabricated cubic sample and e=2 cm is the distance between the stainless steel electrode nets. Using the illustrated electrode arrangement, the variation of resistance, ΔR, induced by the compression load *F* applied in the orthogonal direction (Figure 4a), has been obtained through the equation:

(1) $$R=1000\frac{V_{1}}{V_{2}}$$

where $V_{1}$ is the potential difference through the cubic sample and $V_{2}$ is the potential difference read through the shunt resistor (1 kΩ resistor in Figure 4a). Value for *R* is obtained by selecting the 80% charge points on the biphasic voltage waveforms of $V_{1}$ and $V_{2}$ [33]. The resistivity ρ of the material is obtained using

(2) $$\rho =\frac{A}{e}R$$

where A= 25 cm${}^{2}$ is the cross-sectional area.

The electromechanical tests are conducted in a laboratory under constant temperature conditions. The test setup consists on a voltage input unit, a strain measurement, and voltage reader operated simultaneously. The voltage input and reading is handled by the chassis NI PXIe-1092. A biphasic square wave voltage input of ±2 V, selected to eliminate signal drifts caused by the polarization of cement matrix, is sourced by an NI PXIe-4138 unit. Voltages are read through channels 1 and 2 (ch1 and ch2 in Figure 4) using the 32-channel Analog Input Module NI PXIe-4302 controlled by a NI PXIe-8840 unit. The sampling frequency is set at 10 Hz. The dynamic load F(t) is applied using a testing machine model Advantest 50-C7600 by Controls. The control unit, model 50-C 9842, has a maximum capacity of 15 kN. Induced strains are recorded with three LVDT transducers that allows 10 mm maximum travel distance, placed at 120 degrees in-plane. A preliminary calibration of LVDT transducers was conducted against a 20 mm-long electric strain gauges.

## 4. Experimental Results of Resistivity

**Figure 5:**
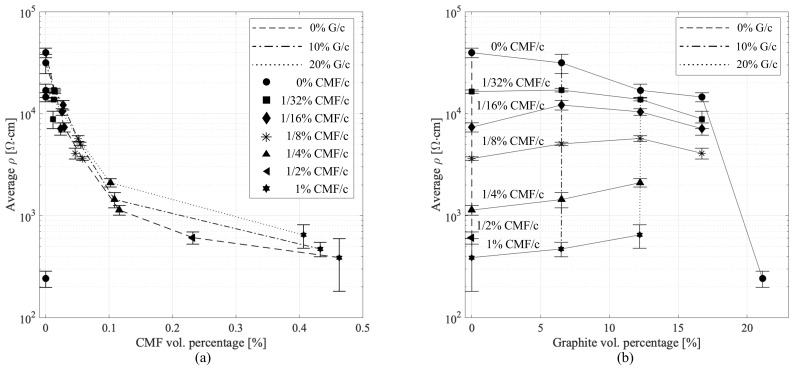
Sample resistivity ρ as a function of doping level showing average values, and the maximum/minimum values denoted by the bar. (**a**) 3D view plotting the evolution of ρ per constant G/c; and (**b**) evolution of ρ per constant CMF/c.

Resistivity measurements were taken 30 days after fabrication on unloaded samples (F(t)=0), following [17] for consistency. Results are plotted in Figure 5, showing the average and the maximum/minimum values under each doping levels arranged by volumetric fractions of G and CMF (Figure 5a).

**Figure 6:**
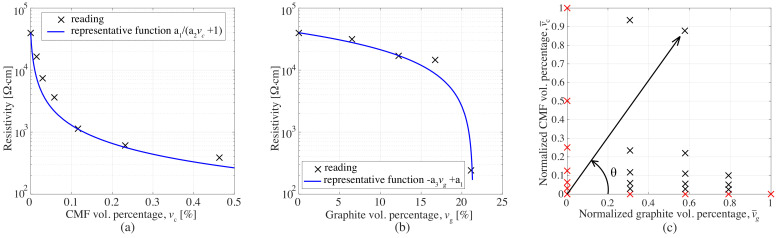
(**a**) Function $f_{c}$ versus CMF-only experimental data; (**b**) function $f_{g}$ versus CMF-only experimental data; and (**c**) polar coordinate system.

It can be observed in Figure 5 that the positive influence of CMF in terms of conductivity on the cement-G matrix is very strong. The inverse correlation of volumetric fraction of CMF and resistivity is also evident (note from Table 2 that for a fixed weight-to-cement ratio of CMF, an increase in volume fraction of graphite results in a decrease in volume fraction of CMF). To explain the observations on an analytical ground, the resistivity has been modeled starting from the boundary conditions of the geometric field defined by the analytical functions $f_{c}$ and $f_{g}$ characterizing the evolution of the resistivity in the CMF-only and G-only samples, respectively:

(3) $$f_{c}\left(\nu \right)=\frac{a_{1}}{a_{2}\nu +1}$$

(4) $$f_{g}\left(\nu \right)=-a_{3}\nu +a_{1}$$

where $a_{i}$ are constants and ν is the volumetric fraction of the inclusions. To establish values for *a*, the volumetric fractions of inclusions were first normalized with respect to their maximum values, yielding the normalized volume fraction ${\bar{v}}_{c}$ for CMF and ${\bar{v}}_{g}$ for G. Figure 6a,b plots the functions $f_{c}$ and $f_{g}$ using a${}_{1}=44,283$, a${}_{2}=126$, and a${}_{3}=44,994$, exhibiting a good fit of experimental data. The tuning of these constants will be discussed later.

**Figure 7:**
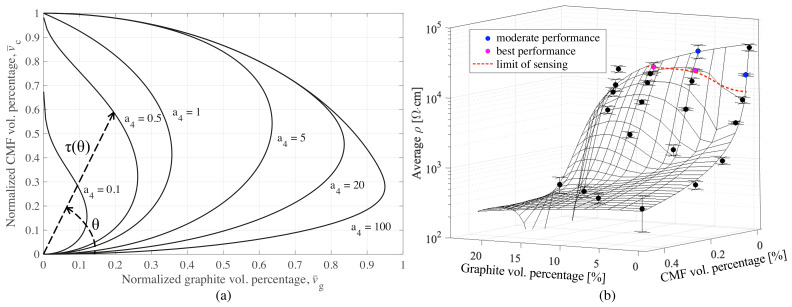
Illustrations regarding to optimization of resistivity surface; (**a**) The distributions of interpolation metric, τ, with respect to changing a${}_{4}$; (**b**) the obtained resistivity surface together with performance evaluation and observed limit of sensing.

The analytical representation of the resistivity as a function of both inclusions was expected to be a nonlinear interpolation function in polar coordinates (*v*,θ) defined in the positive part, where θ is the angle between the line connecting the data point to the origin and the horizontal axis as illustrated in Figure 6c. Here, the extreme value θ=0 denotes G-only samples, and $\theta =\frac{\pi }{2}$ denotes CMF-only samples. The radial component is taken as $v=\sqrt{{\bar{v}}_{c}^{2}+{\bar{v}}_{g}^{2}}$ and the angular component $\theta =\tan^{-1}\left({\bar{v}}_{c}/{\bar{v}}_{g}\right)$. The interpolation is conducted by introducing an angular variable τ(θ). τ(θ) is a continuous function over θ that enables the nonlinearity of the interpolation. The surface function characterizing ρ(v,θ) becomes:

(5) $$\rho \left(v,\theta \right)=\left(1-\tau \left(\theta \right)\right)f_{g}\left(v\right)+\tau \left(\theta \right)f_{c}\left(v\right)$$

with

(6) $$\tau \left(\theta \right)=\frac{\cos^{-1}\left(\cos{\left(\theta \right)}^{a_{4}}\right)}{\pi /2}$$

where a${}_{4}$ is a constant. Figure 7a illustrates how the angular distribution of τ(θ) changes with respect to variable a${}_{4}$. Accordingly, small values of a${}_{4}$ (i.e., below 5) indicates that the significance of G is high, while for greater values influence of CMF becomes more significant. The tuning of the four constants a${}_{i}$ was conducted using a gradient-based multivariable extremum seeking algorithm proposed by Ariyur and Krsti$\overset{´}{c}$ [34], yielding a${}_{1}=44,283$, a${}_{2}=126$, a${}_{3}=44,994$, and a${}_{4}=23$.

Figure 7b shows the resulting surface function with a good fit of the experimental data. On the surface, the best performing samples are indicated with the magenta color. Likewise, sample sets with acceptable performance are indicated in blue. The identification of the performance will be discussed in the next sections. The limit of sensing is determined by experimental observations marked by red line. Beyond that line, the samples exhibit high noise and become nonresponsive to the induced strain in terms of resistance due to overpercolated fillers.

## 5. Strain Sensitivity of Material

**Figure 8:**
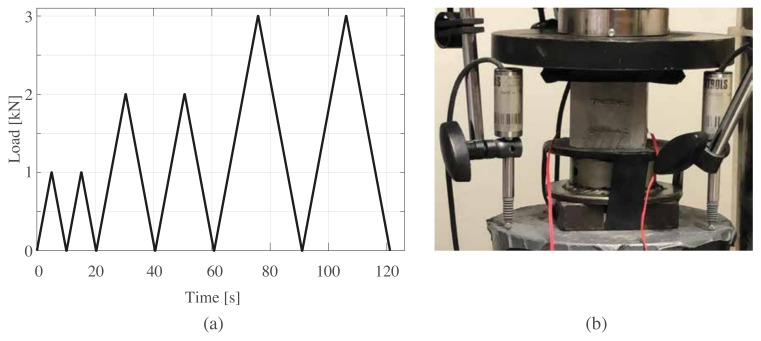
(**a**) The load time history, F(t); (**b**) the setup of the electromechanical tests.

Electromechanical tests were performed on samples 100 days after casting. The test setup is illustrated in Figure 8a, showing the triangular loading pattern at 1, 2, and 3 kN, corresponding to 0.4, 0.8, and 1.2 MPa, respectively (Figure 8a). Figure 8b shows the experimental setup showing a cubic sample instrumented with strain gauges installed and connected to the data acquisition system.

**Figure 9:**
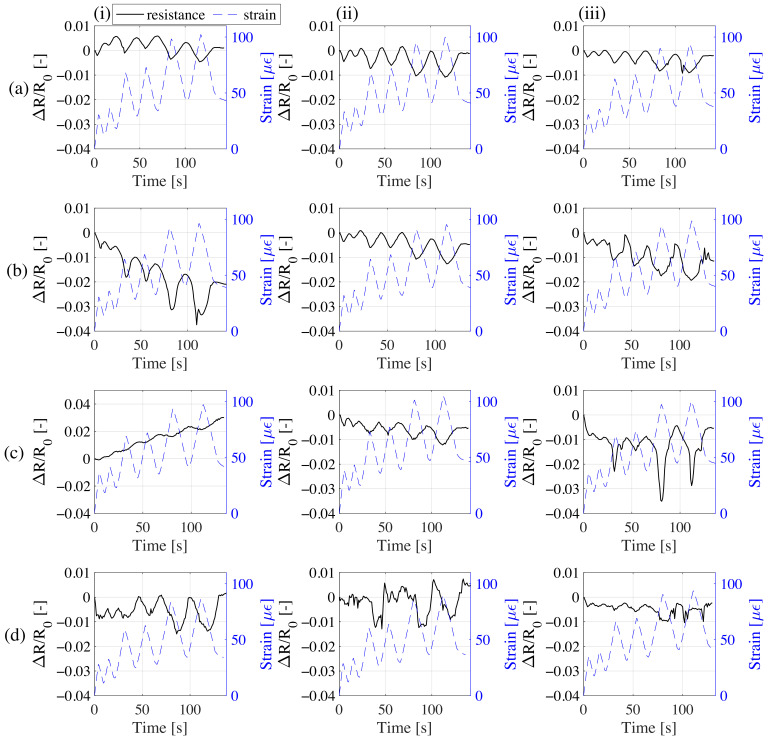
Relative change of resistance versus strain time histories each of the three samples belonging to the set of; (**a**) 1-32CMF0G; (**b**) 1-32CMF10G; (**c**) 1-32CMF20G; (**d**) 1-32CMF30G.

Figure 9 reports the plots of the results obtained on the 1/32% CMF samples. Samples with CMF doping higher than 1/32% CMF/c did not exhibit significantly measurable changes in responses. This can be attributed to the materials being overpercolated. Electrical results are to be compared with the outcomes of the measurements on 100th day graphite-only doped samples tested under the same load history [17].

**Figure 10:**
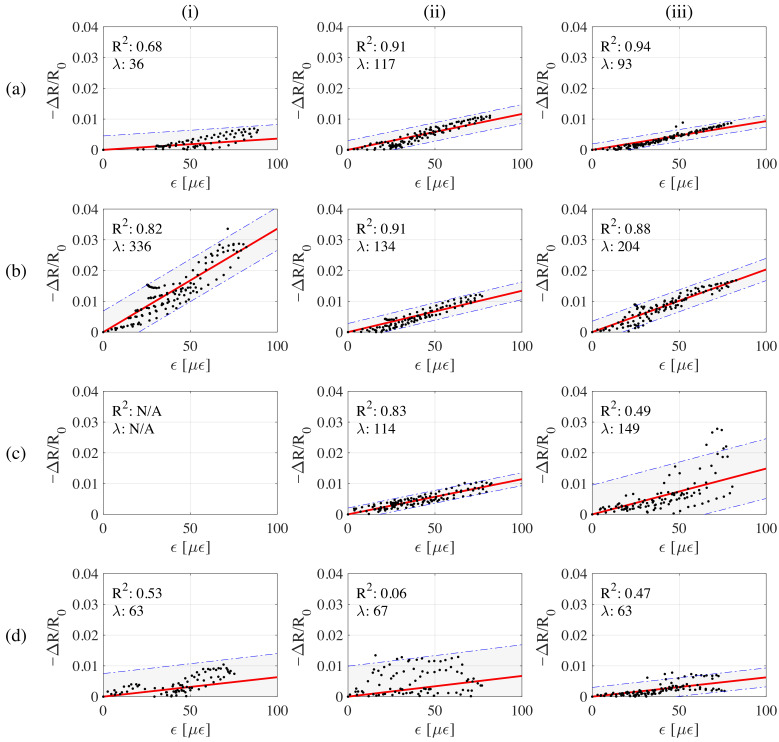
Discrete data points of fractional change of resistance vs. change of strain time histories with the best fitted line for all the samples; (**a**) 1-32CMF0G; (**b**) 1-32CMF10G; (**c**) 1-32CMF20G; (**d**) 1-32CMF30G.

Results from the signals show that samples 1-32CMF10G exhibit the best signal quality in terms of polarization-drift and noise. It can also be observed that samples 1-32CMF0G have good strain sensing performance, but one sample of the set exhibits a noticeable drift due to polarization affecting the reliability of such a material. Signals from samples 1-32CMF20G appear to be unreliable, likely because of instantaneous distortions (Figure 9c(iii)) and polarization ((Figure 9c(i)) caused by the increased dielectric of the overpercolated material. Samples 1-32CMF30G show a similar behavior. Figure 10 presents the sensitivity analysis showing the linearity and gauge factors of the different samples plotted together with 95% confidence fit intervals. Table 3 lists key performance metrics for the samples together with the graphite-only doped samples, where outliers have been discarded after being identified by their negative R${}^{2}$ values. The presented metrics are gauge factor, λ, coefficient of variation of λ, $\sigma_{\lambda }/\mu_{\lambda }$, resolution, δ, related to the 95% confidence interval on the strain domain, and the coefficient of determination, R${}^{2}$. The first load cycle is discarded from the sensitivity analysis to obtain more reliable results without being affected by the initial settlement of the material.

Results demonstrate that the 1-32CMF10G samples exhibit relatively larger gauge factors among all the samples, thus occurring in the transition zone of electrical percolation. It is worth noting that λ of 1-32CMF10G exhibits volatility. After excluding the gauge factor of the less reliable sample (R${}^{2}=0.82$) presented in Figure 10c(i), the average λ of 1-32CMF10G becomes 169 and linear fit has R${}^{2}$ value of 0.89. Therefore, their sensing performance is found comparable to the one of 20% g/c sample. Moreover, the performance evaluation based on resolution (δ), as presented in the Table 3, also points out that most reliable results are those of 1-32CMF10G hybrid and 20% g/c sample sets. After normalization to their maximum observed strain, their normalized resolutions become 0.49 and 0.47, respectively. That indicates these sensors are more precise among others. Compared to the plain cement paste, the presence of CMF increases the linearity of samples considerably. In particular, the performance samples 1-32CMF0G appear to be comparable to 10% G/c graphite only samples; however, the average resolution of 1-32CMF0G set is affected by polarization drift. Finally, 10% G/c and 1-32CMF0G hybrid set possess a moderate good performance, as presented in Figure 7b, where best performing samples are observed to be very close to the limit of sensing line. In addition, the placement of moderately performing samples with respect to the best performing ones and limit of sensing line is found consistent.

The proposed usage of hybrid carbon fillers with the combination of carbon microfibers and graphite powder resulted in notable improvements with respect to the base materials using only microfibers or only graphite doping. The use of a small quantity of carbon microfibers allows reducing the optimum amount of graphite powder from 20% to 10% G/c ratio, which is very beneficial for the workability and mechanical durability of the composite material. Another important remark is that the voltage used for strain reading has been set by progressively reducing voltage below 10 V until a minimum signal drift in time and an optimal noise level were reached. This optimal voltage input for hybrid samples has been found equal to 2 V that, if compared to 10 V as obtained in previous studies on graphite-only inclusions [17], represents a significant improvement facilitating field applications.

## 6. Conclusions

In this study, different combinations of carbon microfibers and graphite are used to produce novel strain sensing smart materials for large scale monitoring applications. Electrical resistivity is found to be accurately described as a nonlinear surface as a function of inclusion levels. The analysis of such a surface clearly demonstrates that graphite percolation is highly accelerated by carbon microfibers. Moreover, higher signal linearity is achieved with the presence of both carbon microfibers and graphite. The best performing hybrid samples are the ones doped with 1/32% CMF/c carbon microfibers + 10% G/c graphite. It is also worth noting that the performance assessment of the samples is supported by the analytical field of resistivity. In terms of power consumption, the optimum voltage level for strain sensing is also reduced to 2V with hybrid fillers, instead of 10 V as needed for samples made of cement-graphite only. The results of the research demonstrate that a good sensing performance is achievable for composites with less amounts of hybrid fillers and lower applied voltage with respect to other investigated composites. Further developing the technology may lead to reduced costs, easier dispersion, increase of workability, simpler data acquisition, and thus facilitate field deployments.

## Figures and Tables

**Table 1:** Materials properties. Aspect ratios are adopted from literature [17] and calculated from [32] by dividing the length of fiber by its diameter.

	Density [g/cm${}^{3}$]	Conductivity [S·cm]	Aspect Ratio [-]
water	1.0	5 × 10${}^{-2}$	N/A
dry cement	1.5	N/A	N/A
G (powder)	1.2	2–3 × $10^{3}$	≈12 [17]
CMF	1.8	6.5 × 10${}^{4}$	≈1000 [32]

**Table 2:** Different samples fabricated for the study, including their constitution.

Sample	Cement (g)	CMF (g)	CMF/c [%]	CMF *v* [%]	G (g)	G/c [%]	G *v* [%]	w/c
0CMF0G	636	0.0	0.00000	0.000	0	0	0	0.50
0CMF10G	636	0.0	0.00000	0.000	64	10	6.7	0.50
0CMF20G	636	0.0	0.00000	0.000	127	20	12.2	0.50
0CMF30G	636	0.0	0.00000	0.000	191	30	17.3	0.55
0CMF40G	636	0.0	0.00000	0.000	254	40	21.8	0.55
1-32CMF0G	636	0.2	0.03125	0.015	0	0	0	0.50
1-32CMF10G	636	0.2	0.03125	0.014	64	10	6.7	0.50
1-32CMF20G	636	0.2	0.03125	0.013	127	20	12.2	0.50
1-32CMF30G	636	0.2	0.03125	0.012	191	30	17.3	0.55
1-16CMF0G	636	0.4	0.06250	0.029	0	0	0	0.50
1-16CMF10G	636	0.4	0.06250	0.027	64	10	6.7	0.50
1-16CMF20G	636	0.4	0.06250	0.026	127	20	12.2	0.50
1-16CMF30G	636	0.4	0.06250	0.023	191	30	17.3	0.55
1-8CMF0G	636	0.8	0.12500	0.058	0	0	0	0.50
1-8CMF10G	636	0.8	0.12500	0.054	64	10	6.7	0.50
1-8CMF20G	636	0.8	0.12500	0.051	127	20	12.2	0.50
1-8CMF30G	636	0.8	0.12500	0.046	191	30	17.3	0.55
1-4CMF0G	636	1.6	0.25000	0.116	0	0	0	0.50
1-4CMF10G	636	1.6	0.25000	0.109	64	10	6.7	0.50
1-4CMF20G	636	1.6	0.25000	0.102	127	20	12.2	0.50
1-2CMF0G	636	3.2	0.50000	0.232	0	0	0	0.50
1CMF0G	636	6.4	1.00000	0.463	0	0	0	0.50
1CMF10G	636	6.4	1.00000	0.433	64	10	6.7	0.50
1CMF20G	636	6.4	1.00000	0.407	127	20	12.2	0.50

**Table 3:** Average results on 0 and 1/32% CMF/c samples.

CMF/c [%]	G/c [%]	λ [-]	$\sigma_{\lambda }/\mu_{\lambda }$	δ [μϵ]	R${}^{2}$ [%]
0	0	60	1.28	3404	57
	10	82	0.44	43	84
	20	183	0.15	19	84
1/32	0	82	0.41	114	84
	10	225	0.37	39	87
	20	131	0.13	82	66
	30	64	0.03	209	36

## Data Availability

The data that support the findings of this study are available from the corresponding author, F.U., upon reasonable request.
